# Ancestral mesodermal reorganization and evolution of the vertebrate head

**DOI:** 10.1186/s40851-015-0030-3

**Published:** 2015-11-09

**Authors:** Takayuki Onai, Toshihiro Aramaki, Hidehiko Inomata, Tamami Hirai, Shigeru Kuratani

**Affiliations:** Kuratani Evolutionary Morphology Laboratory, RIKEN Center for Developmental Biology, 2-2-3 Minatojima-minamimachi, Chuo-ku, Kobe, 650-0047 Japan; Pattern Formation Group, Graduate School of Frontier Biosciences, Osaka University, 1-3 Yamadaoka, Suita, Osaka 565-0871 Japan; Laboratory for Axial Pattern Dynamics, RIKEN Center for Developmental Biology, 2-2-3 Minatojima-minamimachi, Chuo-ku, Kobe, 650-0047 Japan

**Keywords:** Amphioxus, Head mesoderm, Vertebrate body plan, Somites

## Abstract

**Introduction:**

The vertebrate head is characterized by unsegmented head mesoderm the evolutionary origin of which remains enigmatic. The head mesoderm is derived from the rostral part of the dorsal mesoderm, which is regionalized anteroposteriorly during gastrulation. The basal chordate amphioxus resembles vertebrates due to the presence of somites, but it lacks unsegmented head mesoderm. Gastrulation in amphioxus occurs by simple invagination with little mesodermal involution, whereas in vertebrates gastrulation is organized by massive cell movements, such as involution, convergence and extension, and cell migration.

**Results:**

To identify key developmental events in the evolution of the vertebrate head mesoderm, we compared anterior/posterior (A/P) patterning mechanisms of the dorsal mesoderm in amphioxus and vertebrates. The dorsal mesodermal genes *gsc*, *bra*, and *delta* are expressed in similar patterns in early embryos of both animals, but later in development, these expression domains become anteroposteriorly segregated only in vertebrates. Suppression of mesodermal involution in vertebrate embryos by inhibition of convergence and extension recapitulates amphioxus-like dorsal mesoderm formation.

**Conclusions:**

Reorganization of ancient mesoderm was likely involved in the evolution of the vertebrate head.

**Electronic supplementary material:**

The online version of this article (doi:10.1186/s40851-015-0030-3) contains supplementary material, which is available to authorized users.

## Introduction

How the highly complex vertebrate head—composed of brain, head muscles, and skull—evolved from non-vertebrate ancestors is a fundamental question in current evolutionary and developmental biology [1–3]. Recent comparative studies of the cephalochordate amphioxus and vertebrates suggest that a region homologous to the vertebrate fore/mid/hind brain is also present in the rostral part of the central nervous system (CNS) of amphioxus [4–6]. Amphioxus is a basal chordate that has somites extending to the rostral end of the body, and is considered the best proxy for understanding the origin of the vertebrate body plan [7].

The homology between amphioxus and the vertebrate CNS indicates that the unsegmented vertebrate head mesoderm evolved directly from the rostral somites of amphioxus [8]. Expression of *en* in the rostral somites of amphioxus (*Branchiostoma floridae*) and *en2* in the ventral part of the mandibular head mesoderm of shark (*Scyliorhinus torazame*) embryos supports this hypothesis [9, 10]. However, Bf*pax3*/*7*, a homologue of *pax3* that serves as a somite marker in vertebrates, is expressed in the rostral somites, suggesting that the vertebrate head mesoderm did not evolve by simple modification of rostral somites of an amphioxus-like ancestor, but rather by fundamental reorganization that occurred in the dorsal mesoderm [2, 10].

During embryogenesis, the vertebrate head mesoderm derived from the rostral part of the dorsal mesoderm is regionalized along the A/P axis by a gradient of Wnt/β-catenin signalling [11, 12]. In the regionalization of the dorsal mesoderm, downstream regional marker genes of Wnt/β-catenin signalling are expressed in the progenitor domains; *gsc* is expressed in the head mesoderm, whereas *bra* is expressed in the presumptive notochord during the late-gastrula stage [13, 14]. Additionally, in the trunk mesoderm, *delta* expression is detected in the presumptive somite region [15, 16]. Previous functional studies have shown that overexpression of *Xenopus laevis dkk1* (negative regulator of Wnt/β-catenin signalling) expands the *gsc* expression domain posteriorly in *Xenopus* embryos, whereas *bra* expression is activated by Wnt/β-catenin signalling [11, 17, 18]. Additionally, *delta* has an essential role in somitogenesis, and is under the control of Wnt/β-catenin signalling [19].

In amphioxus, *gsc* and *bra* are co-expressed in the presumptive notochordal region at the gastrula stage [20, 21]. The presumptive somite marker *delta* is expressed in the first and second somites in the late-gastrula stage [22]. Loss of *gsc* expression in the notochord and gain of *gsc* expression in the head mesoderm of vertebrates compared with amphioxus indicates that A/P re-arrangement of mesodermal gene expression occurred in the lineage of vertebrates. Excessive Wnt/β-catenin signalling in amphioxus embryos induced by inhibition of GSK-3α/β does not affect the expression of regional marker genes of the dorsal mesoderm, such as *bra* and *fgf8*/*17*/*18*, during the gastrula stage [23]. This suggests that, unlike in vertebrates, Wnt/β-catenin signalling does not play a role in dorsal mesoderm regionalization in amphioxus. If vertebrate embryos did evolve a rearrangement of gene expression in the dorsal mesoderm to generate the head mesodermal region, what was the key developmental event in this process? We consider that rearrangement of gene expression in the vertebrate dorsal mesoderm from an ancestral chordate evolved through a novel mesodermal cell movement present in vertebrates.

Amphioxus gastrulation occurs through simple invagination, with little mesodermal involution of the outer layer [24], whereas in vertebrates, an overt involution takes place, as observed in *Xenopus* and lamprey [25–27]. Thus, uniquely in amphioxus and distinct from the case in vertebrates, there is nearly no change in the relative positions of the ectoderm and mesoderm. However, it remains unclear how mesodermal involution affects A/P patterning of the dorsal mesoderm and how this change has led to two distinct types of rostral mesoderm in amphioxus and vertebrates.

To explore the molecular background of vertebrate head mesoderm evolution, we first investigated the developmental stage at which the overall molecular topography of the dorsal mesoderm becomes distinctly different between amphioxus and vertebrates. We also examined whether mesodermal involution is important for dorsal mesoderm regionalization in vertebrate embryos, as a possible developmental factor that gave rise to vertebrate head mesoderm. Finally, we examined whether the genetic program for mesodermal involution is present in amphioxus. We hypothesize that vertebrate head mesoderm evolved from an amphioxus-like ancestral mesoderm through anteroposterior reorganization of the genetic developmental architecture.

## Materials and methods

### Sources of amphioxus, lamprey, and shark embryos

In the summer breeding season, adult amphioxus (*B. floridae*) were collected from Old Tampa Bay, FL, USA. The in vitro fertilization and culturing of the embryos were conducted as described [28]. Adult amphioxus (*Branchiostoma japonicum*) were collected from the ocean near Amakusa Island, Kumamoto, Japan. Adult lampreys (*Lethenteron japonicum*) were collected from the Miomote River in Niigata, Japan and the Shirubetu River in Hokkaido, Japan during the spring breeding season. In vitro fertilization was performed as described previously [29]. Adult sharks (*S. torazame*) were collected from Nakaminato Bay, Ibaraki, Japan in October and maintained in a seawater tank at 16 °C. Eggs were obtained from the adult females and maintained in the seawater tank until they developed to the described stages [30].

### In situ hybridization

Whole-mount in situ hybridization of amphioxus, lamprey, shark, and *Xenopus* embryos was performed as described in previous studies [10, 29, 31]. For section in situ hybridization of *Xenopus* embryos, larval-stage embryos were fixed using MEMFA for 2 h at room temperature then section in situ hybridization was performed as described [32]. For fluorescence in situ hybridization, the protocol used for amphioxus whole-mount in situ hybridization [23] was applied and an antibody (Anti-DIG-POD, Roche, Basel, Switzerland) and TSA system (PerkinElmer, Waltham, MA, USA) were used. Cellmask deep red (Life Technologies, Carlsbad, CA, USA) (1/1000) and DAPI (Invitrogen, Carlsbad, CA, USA) (1/1000) were used to stain the plasma membrane or nuclei. A Zeiss LSM 780 (Zeiss, Jena, Germany) was used to collect confocal images.

### Plasmid construction and gene markers

The sequences of Bf*dkk1*/*2*/*4* [21], Bf*gsc* [21], Bf*bra* [20], Bf*wnt8* [21], Bf*delta* [22], St*dkk1* (KF551566), St*gsc* (KF564642), St*delta* (KF551567), St*bra* (KF551568), and St*wnt8* (KF551569) were amplified by PCR and cloned into a TOPO cloning vector. Xl*dkk1*-pCS2 (NM_001085592), Xl*gsc*-pCS2 (NM_001087809), Xl*delta*–*2*-pCS2 (NM_001086082), Xl*bra*-pSP64 (M77243.1), Xl*wnt8*-pCS2 (NM_001088168), and Xl*myoD*-pCS2 (NM_001085897) were gifts from Dr. Yoshiki Sasai from RIKEN CDB, Japan. Xl*tbx1* (NM_001090445) was amplified by PCR and cloned into the pCR-II TOPO vector. Lj*gsc* (KF551572), Lj*delta* (KF564639), Lj*bra* (AB501127), and Lj*wnt8* (KF551570) were amplified by PCR and cloned into a TOPO cloning vector. Xl*dd1*-myc-pCS2, myc-Xl*dshdelDEP*-pCS2, and Bf*rnd1*-pCS2 were linearized using *Not*I and transcribed using SP6 polymerase (mMessage mMachine, Ambion, Austin, TX, USA).

### *Xenopus* experiments

Embryos were staged according to the Normal Table published by Nieuwkoop and Faber [33]. The mRNA, in 1× modified Barth’s saline, was injected into the embryos using a fine glass capillary tube and a pressure injector (Narishige, Tokyo, Japan). The embryos were then transferred into 0.1× Barth’s saline until further manipulation or harvest. For histological analysis, the embryos were fixed using Bouin’s fixative and then dehydrated and embedded in paraffin. Sections (6-μm-thick) were cut and stained with haematoxylin and eosin. The sequence of the Xl*rnd1*-morpholino antisense oligonucleotide (MO) has been published [34]. For the dorsal marginal zone assay, embryos were dissected at the early-gastrula stage (stage 10) and cultured in 1× low-calcium magnesium Riner’s supplemented with 0.2 % bovine serum albumin until stage 19.

### Immunostaining of amphioxus and *Xenopus* embryos

Early-neurula-stage amphioxus embryos were fixed using 4 % paraformaldehyde in MOPS buffer at 4 °C overnight, and immunocytochemistry was performed as described [35], using a primary antibody against β-catenin (diluted 1:800 for *Xenopus* embryos and 1:400 for amphioxus embryos; C-2206, Sigma, St. Louis, MO, USA), a secondary Alexa Fluor 488-conjugated antibody (1:400, Invitrogen), and DAPI (1:1000, Invitrogen). Whole embryos and histological sections were imaged using a Zeiss LSM 710 confocal microscope and a Zeiss Axio Zoom V16 microscope, respectively.

### Phylogenetic tree analysis

Phylogenetic trees were constructed with MEGA5 software [36] using the maximum-likelihood method with 1000 bootstrap reiterations. All sequences were aligned using Clustal W (http://www.clustal.org/clustal2/).

### Alignment analysis of Flrt3

For Flrt3 protein alignment analysis, we used the multiple alignment tool in Genetyx–Mac version 16.0.7 (http://www.genetyx.co.jp/products/genetyx_mac_16/index.html).

### Accession numbers

The sequences of the novel genes isolated were deposited in GenBank with following accession numbers. Lj*gsc* (KF551572), Lj*delta1* (KF564639), Lj*wnt8* (KF551570), St*dkk1* (KF551566), St*gsc* (KF564642), St*brachyury* (KF551568), St*delta1* (KF551567), and St*wnt8* (KF551569).

## Results and discussion

### Genetic topography of the vertebrate dorsal mesoderm evolved through A/P expression domain shift of amphioxus mesodermal genes

**Fig. 1:**
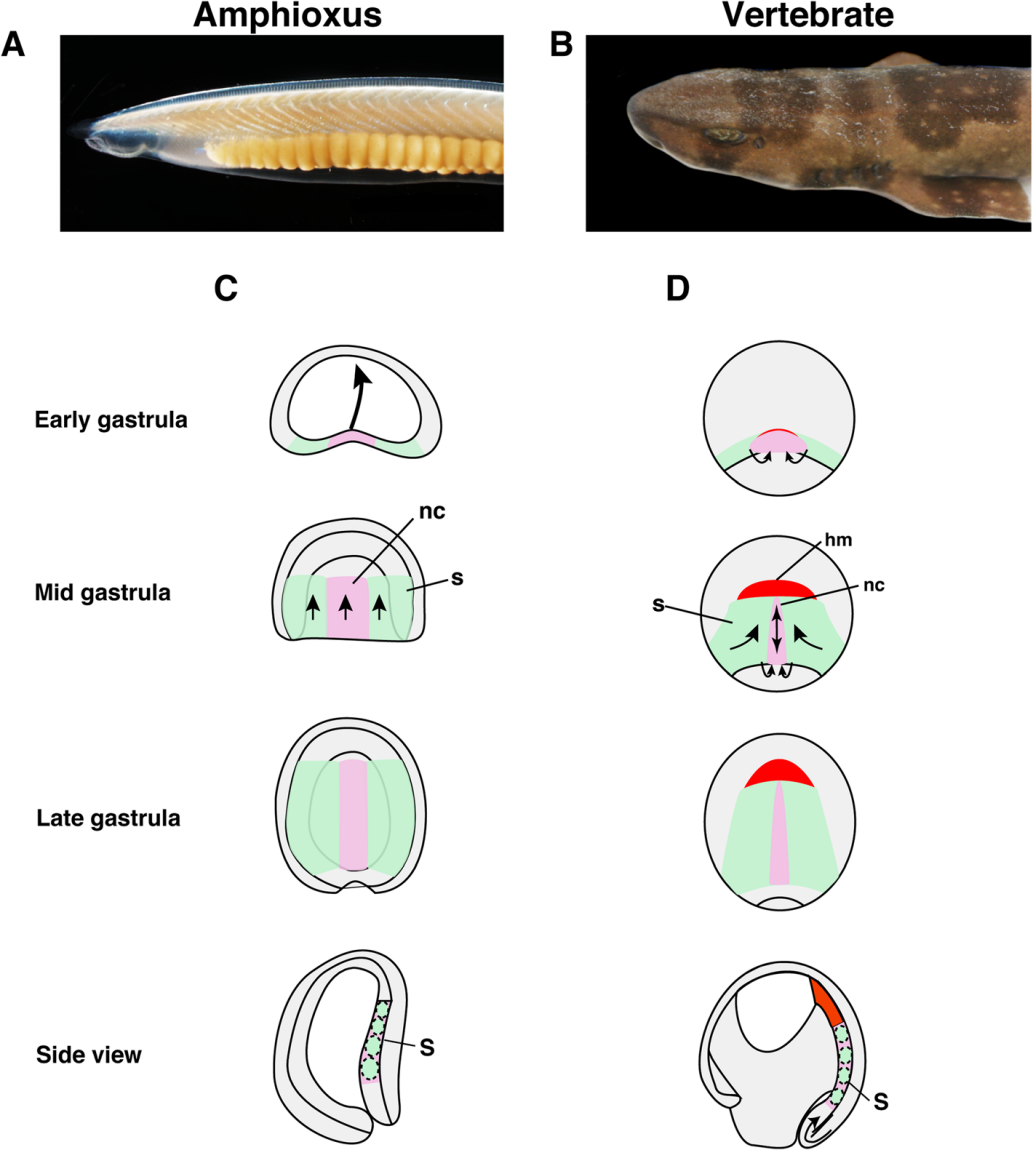
Comparison of amphioxus and vertebrate early development. **a** Adult female amphioxus (*Branchiostoma japonicum*). **b** Adult female shark (*Scyliorhinus torazame*). **c**, **d** Comparison of dorsal mesoderm formation between amphioxus (**c**) and *Xenopus* (**d**) embryos. The notochord progenitor is labelled in *pink*, with the somite in *green* and head mesoderm in *red. nc* notochord, *s* somite, *hm* head mesoderm

In the dorsal mesoderm of amphioxus embryos, somites and notochord are regionalized during gastrulation, and an equivalent structure of the vertebrate unsegmented head mesoderm is thought to be absent in amphioxus [7] (Fig. 1). The dorsal mesoderm of vertebrates such as *Xenopus* is formed by massive mesodermal involution during the early-gastrula stage, which is not present in amphioxus embryos and is regionalized into head mesoderm, notochord, and somites [27, 37] (Fig. 1), suggesting that genetic programs govern differences between amphioxus and vertebrate mesodermal formation. We thus examined the molecular topography of the dorsal mesoderm in amphioxus and *Xenopus*. We first compared the mid-gastrula stage of amphioxus embryos and stage 10.5 *Xenopus* embryos because the orientation of dorsal mesodermal tissue is approximately parallel to the A/P body axis in both species, and thus comparable at these stages [33] (Additional file 1: Figure S1). The key regional genes include *gsc* (head mesoderm), *bra* (notochord), *delta* (somite), *wnt8* (somite) and *dkk1* (head mesoderm, somite).

**Fig. 2:**
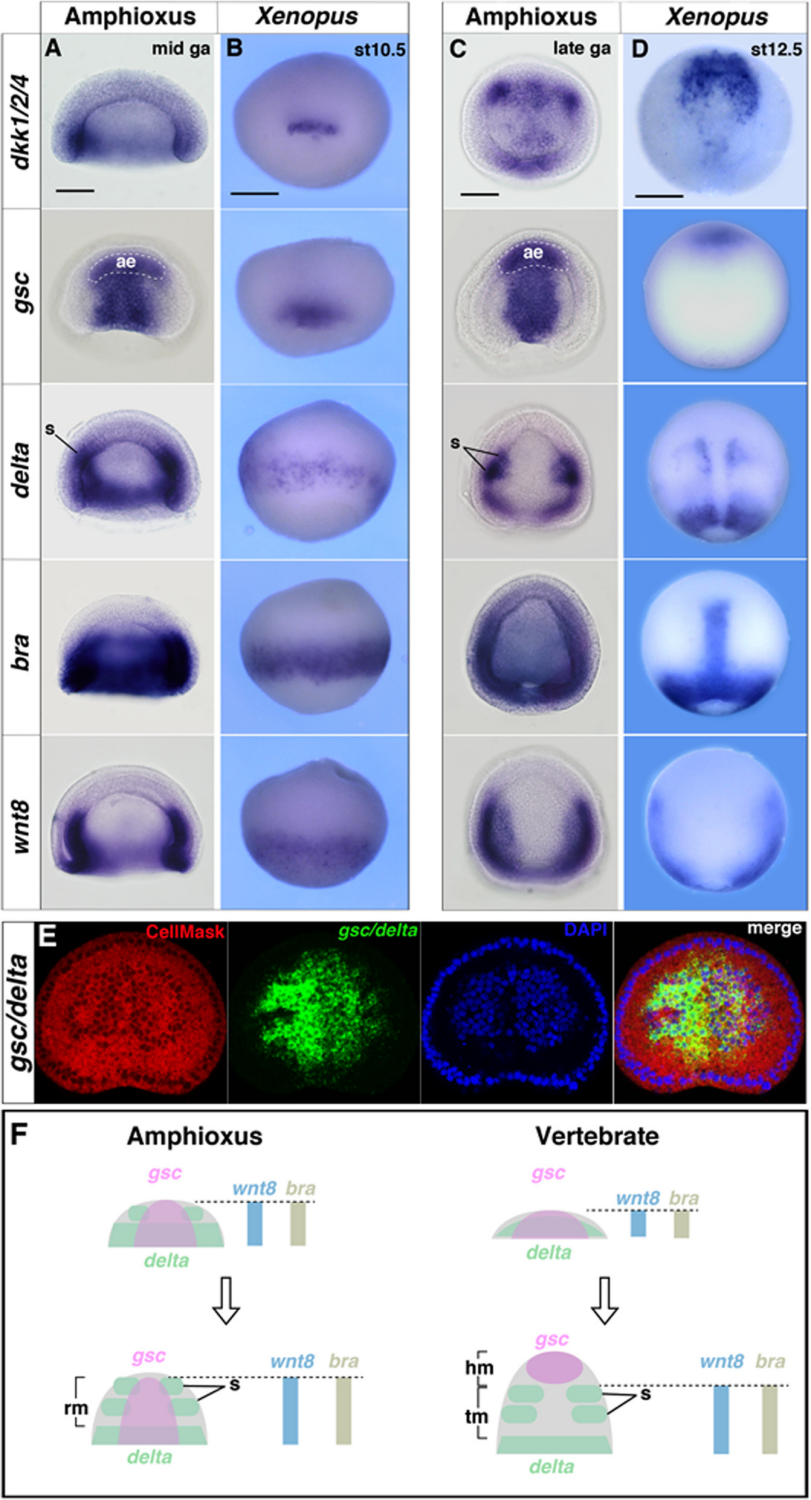
Comparison of regional gene expression patterns in the dorsal mesoderm of amphioxus and vertebrates. **a**–**d** In situ hybridization of (**a**) mid- and (**c**) late-gastrula amphioxus embryos and (**b**) stage 10.5 and (**d**) 12.5 *Xenopus* embryos for *dkk1*/*2*/*4*, *gsc*, *delta*, *bra*, and *wnt8*. Bf*gsc* expression was detected in the anterior endoderm that forms the foregut. *ga* gastrula, *st* stage, *ae* anterior endoderm, *s* somite. **e** Fluorescence in situ hybridization of late gastrula amphioxus embryos stained with *gsc*/*delta* (*Green*). CellMask stained plasma membranes (*red*) and DAPI stained nuclei (*blue*). **f** Summary of dorsal mesoderm gene expression in amphioxus and vertebrates. *Arrows* indicate shifts of developmental stages from early- to late-gastrula stage. *rm* rostral mesoderm, *hm* head mesoderm, *tm* trunk mesoderm. *Scale bars*, 50 μm in amphioxus, 500 μm in *Xenopus*

By the mid-gastrula stage, all genes examined were expressed around the blastopore and showed similar patterns in amphioxus and vertebrates (Fig. 2a and b). However, by the late-gastrula stage of *Xenopus*, the expression domains of the regional marker genes became separated anteroposteriorly, with the *gsc* expression domain barely overlapping with those of *delta*, *wnt8*, and *bra* as the head and trunk mesodermal identities became distinct (Fig. 2c–f). These dynamic shifts were also observed in basal vertebrates, such as the lamprey (*L. japonicum*) and shark (*S. torazame*) (Additional file 1: Figures S2, S3 and Table S1). During the late-gastrula stage, the presomitic mesodermal region became distinct around the blastopore, and *dkk1*/*2*/*4*, *delta*, *bra*, and *wnt8* were co-expressed in *Xenopus* presomitic mesoderm (Fig. 2d). In amphioxus, somites were found to form directly from the tail bud, and the expression of *dkk1*/*2*/*4*, *delta*, *bra*, and *wnt8* largely overlapped in the prospective tail bud region (Fig. 2c). These results suggest that during gastrula stages, the mesodermal genes segregate anteroposteriorly only in vertebrates, whereas in amphioxus, these genes overlap considerably (Fig. 2f).

### Interference of mesodermal involution promotes an amphioxus developmental mode in *Xenopus* embryos

**Fig. 3:**
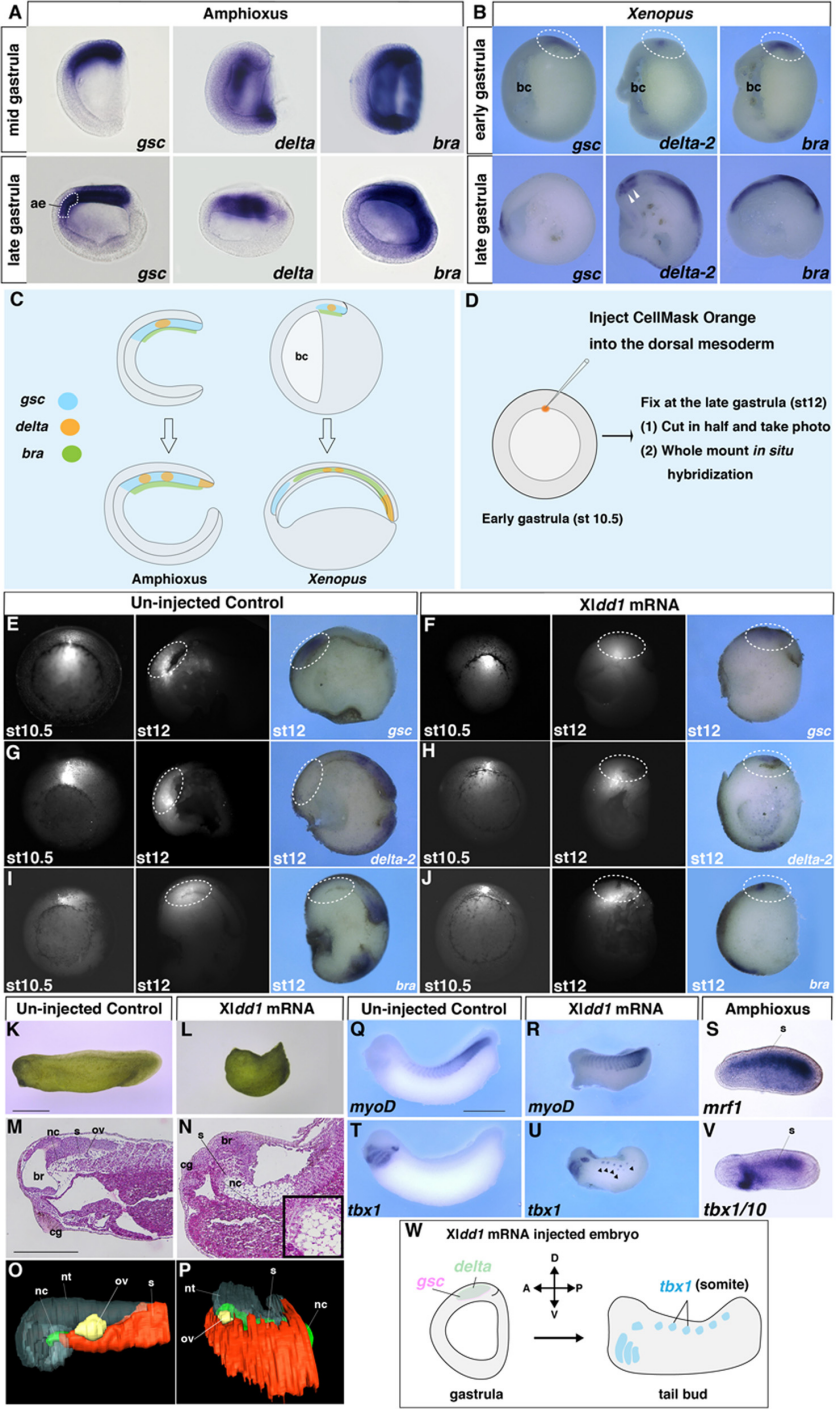
Inhibition of mesodermal involution in vertebrates recapitulates amphioxus A/P patterning. In situ hybridization of *gsc*, *delta* and *bra* in amphioxus (**a**) and *Xenopus* (**b**) embryos. The *arrowheads* for *delta*-*2* in the late gastrula *Xenopus* embryo indicate expression in somites. *ae* anterior endoderm, *bc* blastocoel. **c** Schematic diagram of relative size increase of the dorsal mesoderm during gastrulation in amphioxus and vertebrates. **d** Overview of the labelling study of the dorsal mesoderm in *Xenopus*. CellMask Orange (fluorescent dye) was injected into the dorsal mesoderm at stage (st) 10.5 and the embryos were cultured until stage 12. Fixed embryos were cut in half and then subjected to in situ hybridization. **e**, **g**, **i** At stage 12 in the control, labelled cells migrated anteriorly to form the head mesoderm (*n* = 21, 100 %) and expressed *gsc* (*n* = 2, 100 %) but not *delta*-*2* (*n* = 2, 100 %) or *bra* (*n* = 3, 100 %). **f**, **h**, **j** At stage 12 in the embryos that had been injected with 400 pg/cell Xl*dd1* mRNA at the four-cell blastomere stage, the labelled cells stayed near the blastopore region (*n* = 40, 100 %), and *gsc* (*n* = 2, 100 %), *delta*-*2* (*n* = 6, 100 %), and *bra* (*n* = 3, 100 %) expression overlapped along the A/P axis. *st* stage. The *dotted circle* indicates the cells labelled by CellMask Orange. External morphology (**k**, **l**), histological sections (**m**, **n**) and 3-D reconstruction (**o**, **p**) of control (*n* = 22, 100 %; *left*) and Xl*dd1* mRNA–injected embryos (*n* = 35, 94 %; right). *nc* notochord, *s* somite, *br* brain, *cg* cement gland, *ov* otic vesicle, *nt* neural tube. Expression of *myoD* (muscle differentiation marker) (**q**; *n* = 15, 100 %, **r**; *n* = 14, 100 %) and *tbx1* (**t**; *n* = 12, 100 %, **u**; *n* = 20, 90 %) in control and Xl*dd1* mRNA–injected *Xenopus* embryos, respectively. **s** *mrf1* and (**v**) *tbx1*/*10* (pharynx and somite marker) expression in amphioxus embryos. **w** Schematic diagram of amphioxus-like developmental patterns in Xl*dd1* mRNA–injected *Xenopus* embryos. *Scale bars*, 1 mm (**k**, **q**) and 500 μm (**m**)

Just after the stage in which mesodermal gene expression arrangements were similar in amphioxus and *Xenopus*, the dorsal mesoderm in *Xenopus* spread anteriorly to the blastocoel to form the head mesoderm (Fig. 3a and b). However, in amphioxus, the relative increase in mesodermal size was much lower than that in vertebrates (Fig. 3c). This suggests that, in vertebrates, the dynamic mesodermal gene shift is achieved by increasing the mesoderm, which is primarily dictated by mesodermal cell movements (e.g. involution, convergence and extension) (Fig. 3c). During gastrulation, mesodermal involution is controlled by convergence and extension of the dorsal axial mesoderm in vertebrates [38, 39]. To determine whether mesodermal involution is essential for the mesodermal gene shift, we suppressed convergent extension by inhibiting the Wnt planar cell polarity (PCP) signalling pathway, (a key signal transduction pathway in convergence and extension) [40, 41]. For the loss of function study of Wnt/PCP signalling, we injected Xl*dd1* (a dominant-negative form of Xl*dsh*; [42, 43]) mRNA into *Xenopus* embryos [26].

In the dye only-injected control embryos, labelled cells migrated anteriorly and expressed *gsc* but not *delta*-*2* or *bra* (Fig. 3d, e, g and i). In Xl*dd1* mRNA-injected embryos, however, labelled cells did not migrate anteriorly, but remained close to the blastopore, and *gsc*, *delta*-*2*, and *bra* were not separated anteroposteriorly as observed in the control embryos during the late-gastrula stage (Fig. 3f, h and j). Additionally, the size of the dorsal mesoderm was much smaller in the Xl*dd1* mRNA–injected embryos compared with control embryos (Fig. 3e–j), indicating that the developmental sequences of the dorsal mesoderm were somewhat similar to those in amphioxus. Microinjection of Xl*dshdelDEP* mRNA, a mutant *dsh* that specifically inhibits the Wnt/PCP signalling pathway [44], indicated that the effect of Xl*dd1* injection resulted from suppression of the Wnt/PCP signalling pathway (Additional file 1: Figure S4A–F). Overlapping head and trunk marker gene expression was also detected based on the results of the dorsal marginal zone assay, suggesting that the effect of Xl*dd1* injection was not attributable to differences in the amount of yolk, but more likely to the loss of mesodermal cell movement (Additional file 1: Figure S4I–L).

These results are consistent with morphological changes observed at the tail bud stage in Xl*dd1*-injected *Xenopus* embryos assimilated to an amphioxus-like condition. Specifically, the anterior-most somite, normally appearing just posterior to the anterior end of the notochord, was extended anteriorly into the prechordal domain (Fig. 3k–p). In these embryos, the somite marker *myoD* was expressed normally, similar to *mrf1* in amphioxus embryos (Fig. 3q–s). Interestingly, ectopic expression of *tbx1*, a head mesoderm marker in vertebrates [10], was also detected in the Xl*dd1*-injected *Xenopus* somites, similar to amphioxus *tbx1*/*10*, a somite marker in amphioxus [45], expression (Fig. 3t–w and Additional file 1: Figure S4G and H). These results suggest that the vertebrate-specific mesodermal involution during gastrulation is likely responsible for the A/P distinction between head and trunk mesoderm, which does not occur in amphioxus.

### A/P patterning in the dorsal mesoderm by different Wnt/β-catenin-signalling input is a vertebrate novelty

**Fig. 4:**
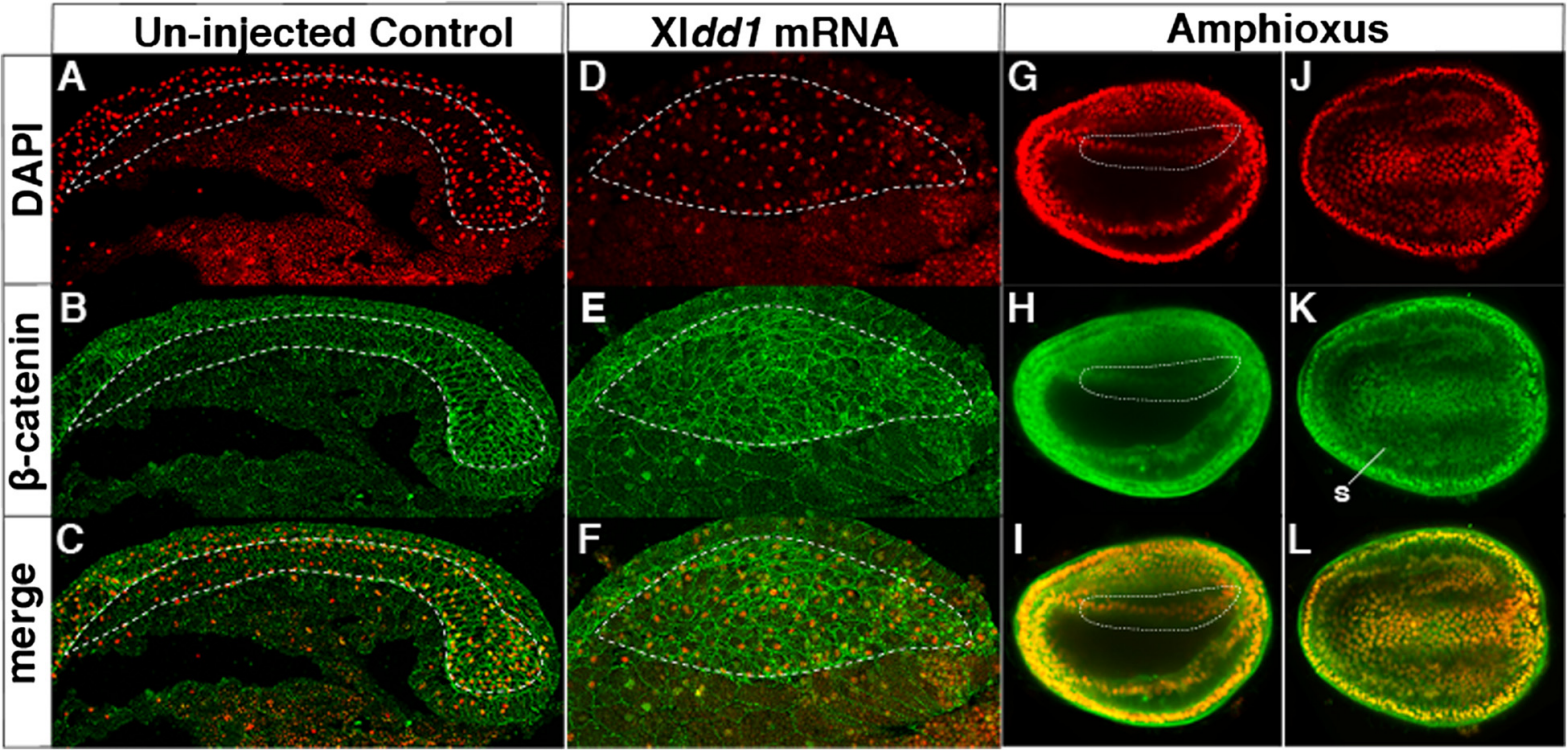
Difference of Wnt/β-catenin-signalling input in the amphioxus and vertebrate dorsal mesoderm. Nuclear β-catenin staining in the *Xenopus* dorsal mesoderm of control embryos (**a**–**c**; *n* = 14, 100 %), embryos injected with 400 pg/cell Xl*dd1* mRNA (**d**–**f**; *n* = 4, 100 %) and in the amphioxus dorsal mesoderm (**g**–**l**); (**g**–**i**) lateral view and (**j**–**l**) dorsal view. Nuclei were stained with DAPI. The *dotted white lines* indicate the dorsal mesoderm. *s* somite

Previous functional studies in vertebrates have shown that the dorsal mesoderm is regionalized by a Wnt/β-catenin-signalling gradient along the A/P axis during early embryogenesis [46]. The failure of A/P segregation of mesodermal regional gene expression in Xl*dd1* mRNA-injected embryos suggests that Wnt/β-catenin-signalling pathway control of these downstream genes is compromised in this context. We examined the nuclear localization of β-catenin, a downstream factor in the Wnt/β-catenin signalling pathway, in Xl*dd1* mRNA-injected embryos. In the control embryos, nuclear localization of β-catenin was observed in the posterior dorsal mesodermal cells, but not in the anterior region (Fig. 4a–c). In Xl*dd1* mRNA-injected embryos, however, β-catenin localized to the nucleus in some cells, but there was no clear A/P difference in the degree of nuclear localization (Fig. 4d–f). The lack of obvious A/P difference in β-catenin nuclear localization was also observed in the amphioxus dorsal mesoderm (Fig. 4g–l). Consistent with this result, a previous functional study showed that Wnt/β-catenin signalling had no role in the A/P patterning of the dorsal mesoderm during the gastrula stages [23]. These findings suggest that anterior low and posterior high Wnt/β-catenin-signalling input is important in the segregation of regional marker genes of the dorsal mesoderm along the A/P axis that evolved in vertebrates.

### Evolution of head mesoderm in vertebrates

**Fig. 5:**
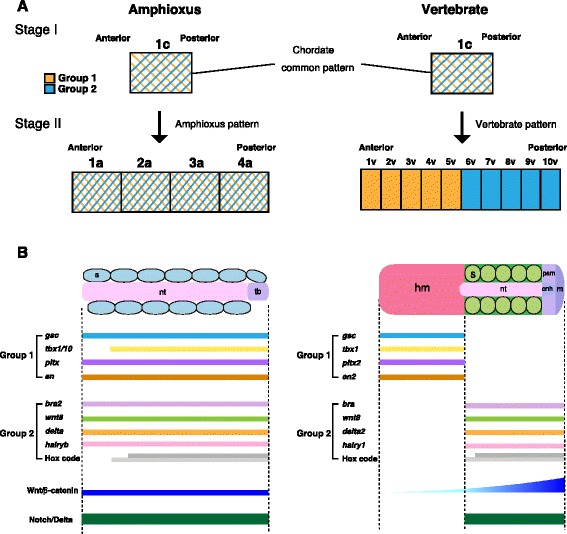
Vertebrate head mesoderm evolved through polarization of ancestral mesodermal patterning. **a** In amphioxus and vertebrates, the dorsal mesoderm has two groups of genes that encode positional value (Group 1, *orange*; Group 2, *light blue*). At stage I (early-to mid-gastrula stages), group 1 and group 2 are co-expressed both in amphioxus and vertebrate dorsal mesoderm. At stage II (late-gastrula stage), group 1 genes shift anteriorly and group 2 genes shift posteriorly in vertebrates, whereas the amphioxus mesoderm retains the overlapped condition. *Numbers* indicate positional value of the dorsal mesoderm along the A/P axis. *c* chordate, *a* amphioxus, *v* vertebrates. **b** The dorsal mesoderm of vertebrates evolved by the reorganization of mesodermal genes under the control of Wnt/β-catenin-signalling input. Notch/Delta signalling regulates somitogenesis both in amphioxus and vertebrates. *s* somite, *nt* notochord, *tb* tail bud, *psm* presomitic mesoderm, *cnh* chordoneural hinge, *m* mesenchyme, *hm* head mesoderm. *hairy* genes are key components that regulate somite formation during development [16]. *pitx2* and *en2* are expressed in head mesoderm of vertebrate embryos [10]

In this study, we propose that the vertebrate dorsal mesoderm evolved as an entirely novel pattern associated with a new mechanism of mesoderm specification. As was first described by Ernst Haeckel [47], amphioxus gastrulation involves a simple invagination similar to that in cnidarians, with little change in the spatial relationships between the ectoderm and mesoendoderm during development. Unlike in vertebrates, the dorsal mesoderm in amphioxus co-expresses both vertebrate head and trunk mesoderm marker genes (Fig. 5). Thus, the amphioxus dorsal mesoderm remains unspecified along the A/P axis due to the absence of a vertebrate-specific developmental program (Fig. 5). Vertebrate mesoderm, however, is uniquely polarized along the A/P axis into the head and trunk mesoderm based on mesodermal patterning mediated by vertebrate-specific cell movement. By the end of gastrulation, the head and trunk mesodermal identities are specified by anteroposteriorly dislocated expression of regional marker genes. This patterning mechanism also controls A/P patterning of the overlying neuroectoderm. In vertebrates, the A/P regional identity of the CNS is organized by vertical signals from the underlying dorsal mesoderm containing A/P pattern information [48]. In amphioxus, based on the topography of regional markers, the CNS is patterned into domains largely comparable to the fore-/mid-/hindbrain and the spinal cord in vertebrate embryos [5]. However, three major signalling centres (anterior neural ridge, zona limitans intrathalamica, and midbrain–hindbrain boundary) are absent in the amphioxus CNS [5, 49]. Given that our current study indicated that the A/P regional identity of the vertebrate dorsal mesoderm is fundamentally different from that of amphioxus (Fig. 5a), the three major signalling centres in the neuroectoderm of vertebrate embryos may have evolved through a reorganization of the dorsal mesoderm in an amphioxus-like chordate ancestor.

Evolutionary reorganization of the entire dorsal mesoderm as described above would imply that individual amphioxus somites are not homologous to any specific region of the vertebrate head mesoderm. Our scenario instead favours the novel nature of the vertebrate mesoderm generated by modification in mesodermal patterning dynamics. From the perspective of vertebrate mesodermal specification, the segmented mesoderm in amphioxus appears as an intermediate between the head mesoderm and trunk somites, and vertebrate somites do not represent primitive traits, but rather derived traits established by removal of head mesoderm-like properties from ancestral somites. This scenario of vertebrate head evolution correlates with the observation that peripheral nerves in amphioxus possess traits of both cranial and spinal nerves [50, 51]. The mesodermal developmental pattern is shared among chordates only in the early gastrulae, in which the mesoderm is not yet anteroposteriorly polarized, possibly representing a plesiomorphic state (Fig. 5). The A/P patterning of mesodermal identities through the different Wnt/β-catenin-signalling input takes place only in vertebrates later in the developmental process; along with unique changes in cell movement, this can be considered a synapomorphic developmental trait for this animal subphylum. As proposed by Haeckel, this indicates that the vertebrate body plan is established by recapitulating an amphioxus-like ancestral pattern (plesiomorphy) during the early-gastrula stage and engaging a novel pattern (synapomorphy) during the later stages. The comparison of mesodermal gene expression of vertebrates, amphioxus, and hemichordates (as an out-group taxon) suggests that the amphioxus mesoderm has an intermediate nature, possibly representing a plesiomorphic state for deuterostomes (http://www.ibiology.org/ibioseminars/evolution-ecology/marc-w-kirschner-part-3.html). This characterization of the evolutionary sequence of developmental dynamics in chordates also provides insight into the potential origins of mesoderm and mesodermal segments in bilaterians.

### Possible mechanism of mesodermal involution unique to vertebrates

In this study, we showed that inhibition of mesodermal involution in vertebrate embryos recapitulated amphioxus development (Fig. 3). The lack of mesodermal involution and likely convergent extension in amphioxus gastrulation indicates that the developmental program for mesodermal involution in vertebrates is absent in amphioxus. Disruption of cadherin-mediated cell–cell adhesion is essential during mesodermal involution and convergent extension in vertebrates, and fibronectin leucine-rich-repeat transmembrane 3 (Flrt3) and a small GTPase (Rnd1) control C-cadherin degradation [26, 34, 52, 53]. A BLAST search revealed a homologue of *rnd1* in amphioxus, but not of *flrt3* (Additional file 1: Figure S5A and B). In amphioxus, *rnd1* expression was observed around the blastopore (Additional file 1: Figure S6A–H). However, overexpression of Bf*rnd1* mRNA could not rescue the loss of endogenous *rnd1* in *Xenopus* (Additional file 1: Figure S6I–M). These findings suggest that involvement of the cadherin degradation system in mesodermal involution as well as convergence and extension may have emerged in the vertebrate lineage.

## Conclusions

Our findings indicate that the A/P patterning of the vertebrate dorsal mesoderm evolved from an amphioxus-like ancestral mesoderm through A/P polarization of mesodermal specification to divide into the unsegmented head mesoderm anteriorly and the segmented trunk somites posteriorly. Vertebrate head mesoderm is thus an evolutionary novelty.

## Additional file

Additional file 1: Figure S1. Dorsal mesoderm formation in chordates. **Figure S2.** Phylogenetic trees of dorsal mesoderm genes. **Figure S3.** Dorsal mesodermal gene expression in lamprey (*L. japonicum*) and shark (*S. torazame*) embryos. **Figure S4.** Suppression of the Wnt/PCP-signaling pathway in *Xenopus* embryos. **Figure S5.** Flrt3 evolved in the vertebrate lineage. **Figure S6.** Flrt3-Rnd1 system is essential for mesoderm formation in vertebrates. **Table S1.** Summary of expression pattern of genes in **Figure S2.** (DOCX 8727 kb)
