# Supplementary material for: Ancestral mesodermal reorganization and evolution of the vertebrate head
Source: Zoological Lett. 2015 Nov 9;1:29. doi: 10.1186/s40851-015-0030-3 (PMC4657371; doi:10.1186/s40851-015-0030-3)
Supplement: Additional file 1: Figure S1. — Dorsal mesoderm formation in chordates. Figure S2. Phylogenetic trees of dorsal mesoderm genes. Figure S3. Dorsal mesodermal gene expression in lamprey (L. japonicum) and shark (S. torazame) embryos. Figure S4. Suppression of the Wnt/PCP-signaling pathway in Xenopus embryos. Figure S5. Flrt3 evolved in the vertebrate lineage. Figure S6. Flrt3-Rnd1 system is essential for mesoderm formation in vertebrates. Table S1. Summary of expression pattern of genes in Figure S2. (DOCX 8727 kb) [file 40851_2015_30_MOESM1_ESM.docx]

**Additional file 1**

**Ancestral mesodermal reorganization and evolution of the vertebrate head**

**Takayuki Onai, Toshihiro Aramaki, Hidehiko Inomata, Tamami Hirai, and Shigeru Kuratani**

**
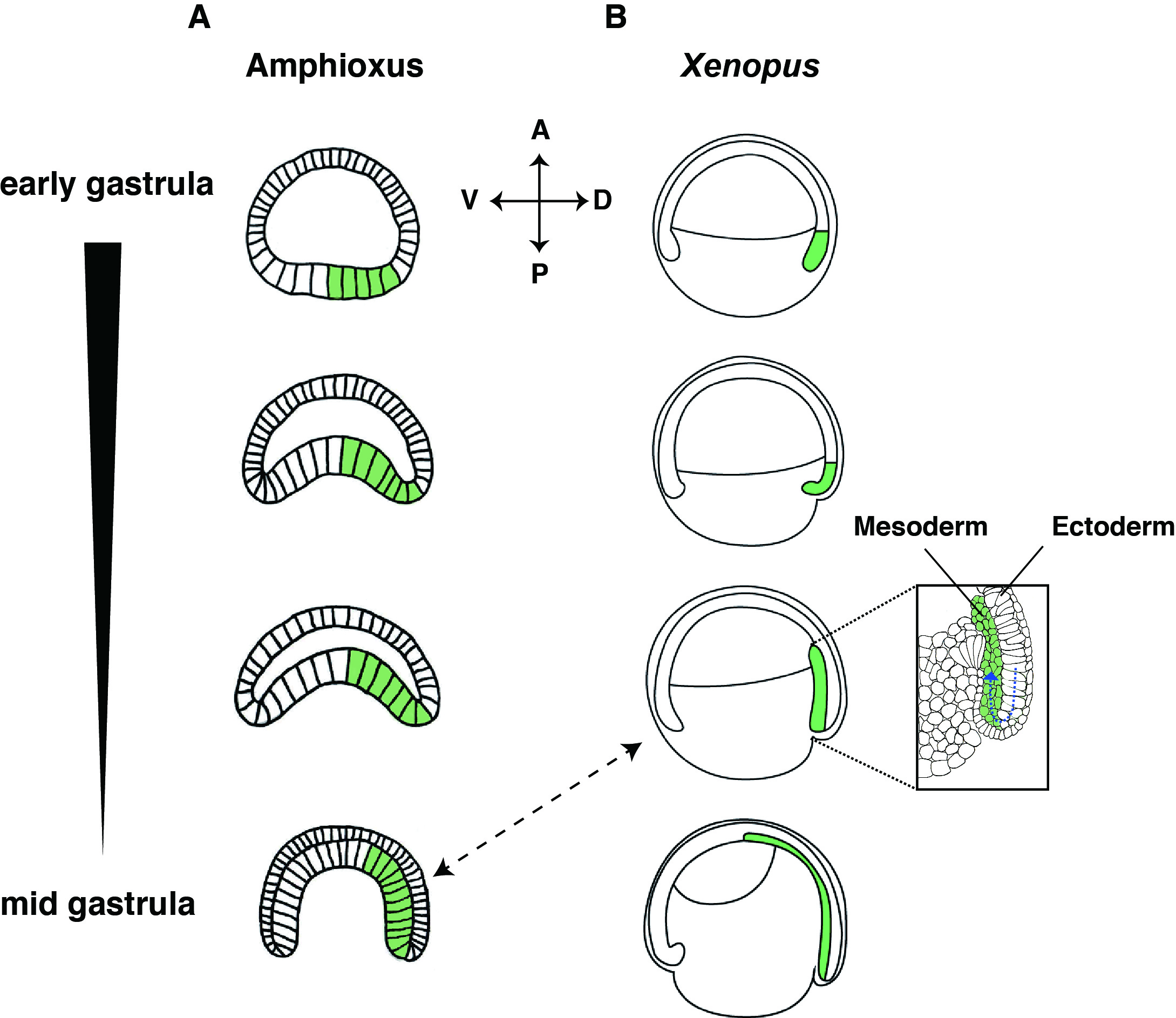
**

Figure S1. Dorsal mesoderm formation in chordates (see Figure 1)

(**A**) During the gastrula stage in amphioxus embryos, mesoderm (green) internalizes by simple invagination, with little involution. (**B**) In *Xenopus*, the dorsal mesoderm involutes from the early-gastrula stage. The dorsal cells internalize to form the dorsal mesoendoderm (blue arrow). Along the A/P axis, the dashed double-headed arrow indicates the initial stages that are comparable between amphioxus and *Xenopus*.

**
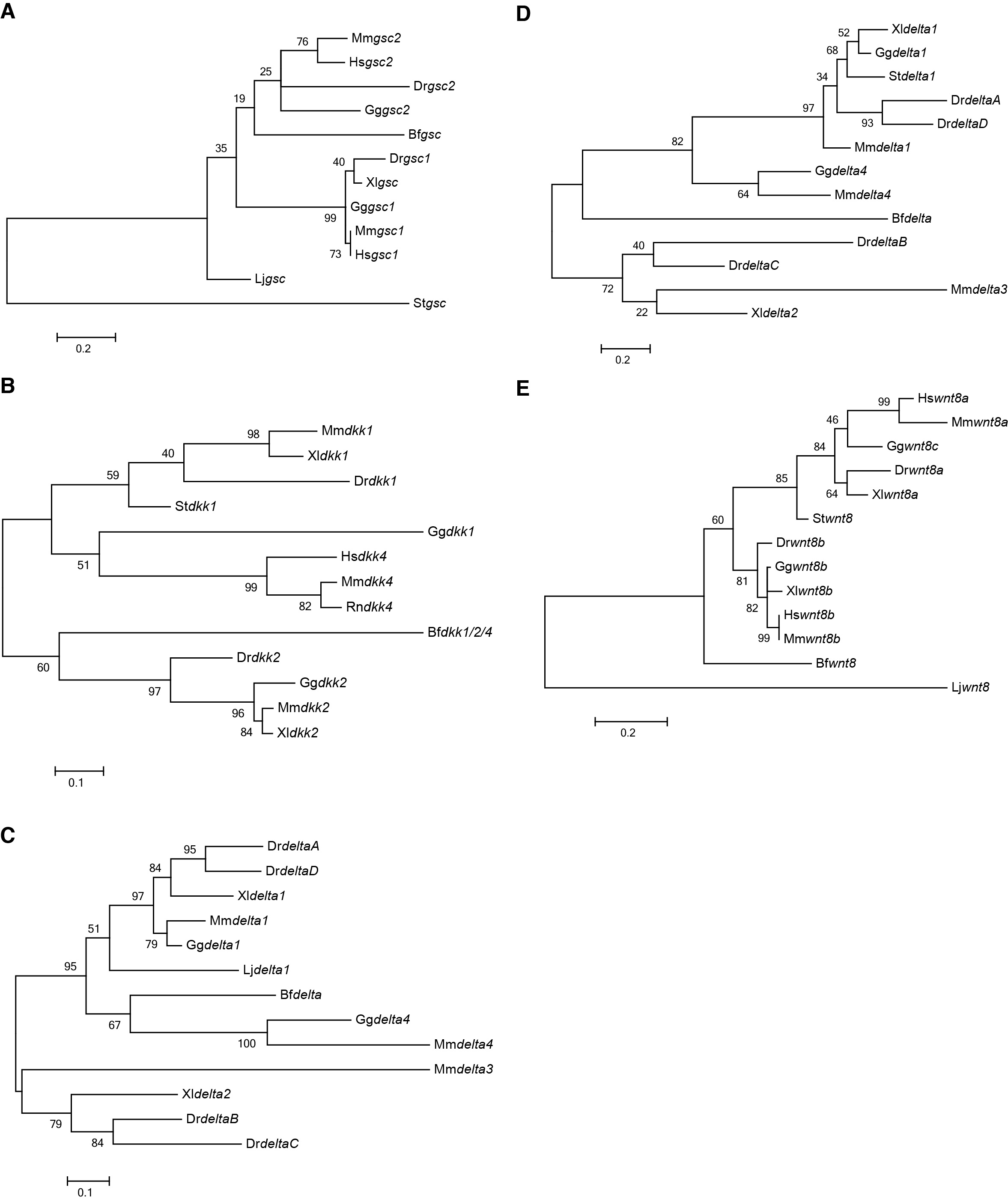
**

Figure S2. Phylogenetic trees of dorsal mesoderm genes (see Figure 2). All trees were estimated using the maximum-likelihood method with 1000 bootstrap values. (**A**) Phylogenetic tree of *Gsc* genes: Mm*gsc2* (AAI25357), Hs*gsc2* (NM_005315), Dr*gsc2* (XP_005171782), Gg*gsc2* (XP_004934385), Bf*gsc* (AF281674), Dr*gsc1* (NM_131017), Xl*gsc* (M81481), Gg*gsc1* (NM_205331), Mm*gsc1* (NM_010351), Hs*gsc1* (AAB31968), St*gsc* (KF564642), and Lj*gsc* (KF551572). (**B**) Phylogenetic tree of *dkk* genes: Mm*dkk1* (AAC02426), Xl*dkk1* (ABM83148), Dr*dkk1* (BAA82135), St*dkk1* (KF551566), Gg*dkk1* (XP_421563), Hs*dkk4* (BAA33475), Mm*dkk4* (NP_663567), Rn*dkk4* (NP_001102802), Bf*dkk1/2/4* (ABG34307), Dr*dkk2* (BAN10328), Gg*dkk2* (XP_420494), Mm*dkk2* (CAB60110), and Xl*dkk2* (ADR82889). (**C**, **D**) Phylogenetic trees of *Delta* genes: Dr*deltaA* (ABL14114), Dr*deltaD* (AF426384_1), Xl*delta1* (AAC38017), Mm*delta1* (AAR30869), Gg*delta1* (NP_990304), Lj*delta1* (KF564639), Bf*delta* (XM_002590025), Gg*delta4* (XM_421132), Mm*delta4* (BC049130), Mm*delta3* (NM_007866), Xl*delta2* (NM_001086082), Dr*deltaB* (AAH76414), Dr*deltaC* (AAI62095), and St*delta1* (KF551567). (**E**) Phylogenetic tree of *wnt8* genes: Hs*wnt8a* (NP_490645), Mm*wnt8a* (NP_033316), Gg*wnt8c* (BAD95607), Dr*wnt8a* (NP_571021), Xl*wnt8a* (NP_001081637), St*wnt8* (KF551569), Dr*wnt8b* (NP_571034), Gg*wnt8b*, (XP_426508), Xl*wnt8b* (AAI69891), Hs*wnt8b* (NP_003384), Mm*wnt8b* (NP_035850), Bf*wnt8* (AF190470_1), and Lj*wnt8* (KF551570). Mm, *Mus musculus*; Hs, *Homo sapiens*; Dr, *Danio rerio*; Gg, *Gallus gallus*; Bf, *Branchiostoma floridae*; Xl, *Xenopus laevis*; St, *Scyliorhinus torazame*; Lj, *Lethenteron japonicum*; Rn, *Rattus norvegicus*; sequences with accession numbers were obtained from the National Center for Biotechnology Information (NCBI; http://www.ncbi.nlm.nih.gov).

**
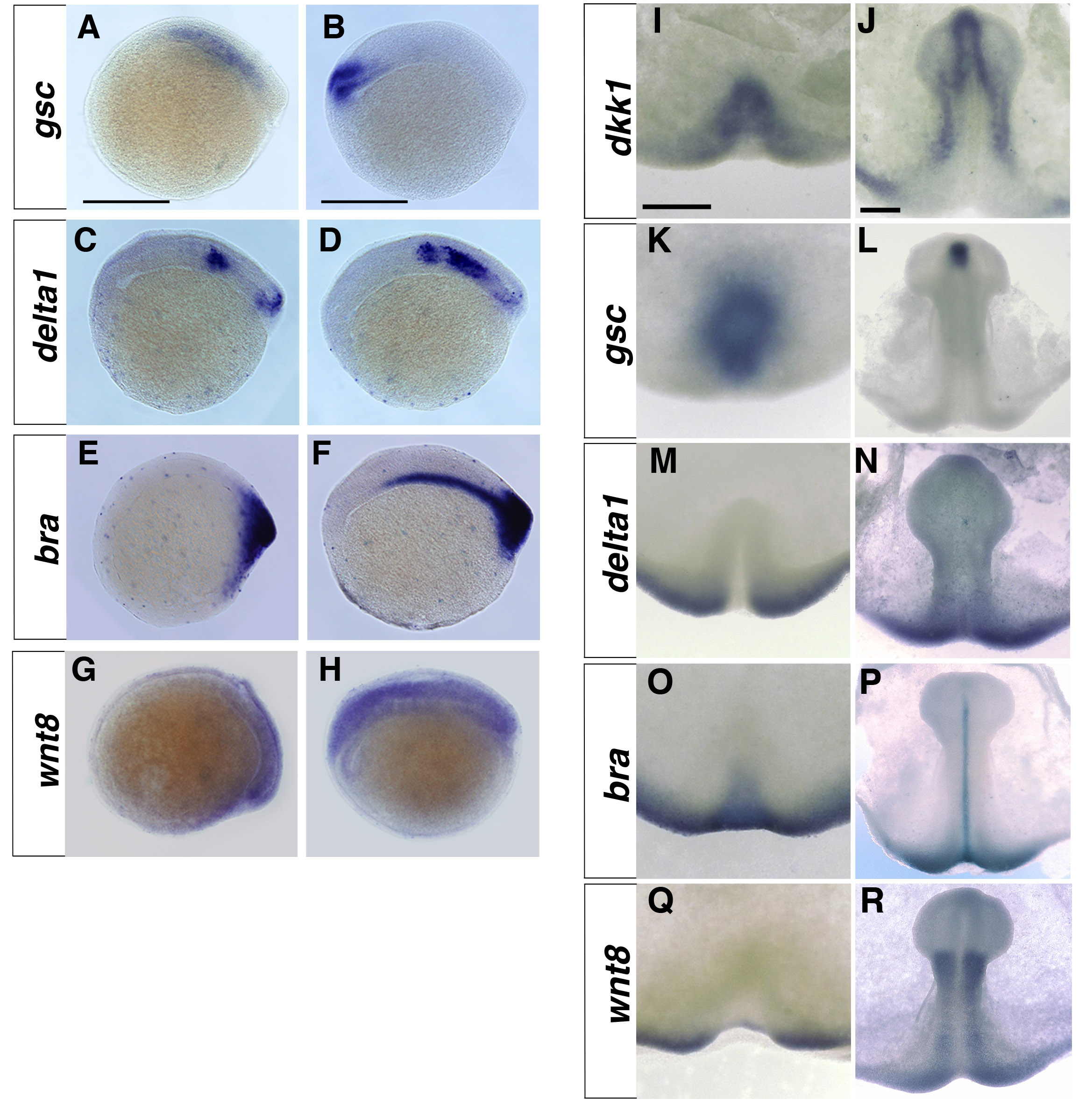
**

Figure S3. Dorsal mesodermal gene expression in lamprey *(L. japonicum*) and shark (*S. torazame*) embryos (see Figure 2) (**A**, **B**) Expression of *gsc* in lamprey; *gsc* was expressed in the mid to trunk dorsal mesoderm at stage 16 (**A**) and in the prechordal plate at stage 18 (**B**). (**C**, **D**) Expression of *delta1* in lamprey; *delta1* was expressed in the trunk region from stages 16 (**C**) to 18 (**D**). (**E**, **F**) Expression of *bra* in lamprey; *bra* was expressed around the blastopore and posterior end of the dorsal mesoderm at stage 16 (**E**). At stage 18, the *bra* expression domain was detected in the notochord and blastopore (**F**). (**G**, **H**) Expression of *wnt8* in lamprey; *wnt8* was expressed in the posterior part of the dorsal mesoderm at stage 16 (**G**). At stage 18, *wnt8* was expressed in the pan-dorsal mesoderm (**H**). (**I**, **J**) Expression of *dkk1* in shark embryos; *dkk1* was expressed in the mesoderm in the early gastrula (**I**) and by the early neurula stage, *dkk1* was expressed in the prechordal plate, posterior region of the paraxial head mesoderm and somites (**J**). (**K**, **L**) Expression of *gsc* in shark. The expression domain of *gsc* was observed around the blastopore at the early-gastrula stage (**K**), whereas at the early-neurula stage, *gsc* was expressed in prechordal plate mesoderm (**L**). (**M**, **N**) Expression of *delta1* in shark. During the early-gastrula stage, *delta1* was expressed in the posterior domain of the dorsal mesoderm (**M**). By the early-neurula stage, expression was expanded anteriorly where the forming somites lined up (**N**). (**O**, **P**) Expression of *bra* in shark. The domain of *bra* expression was observed around the blastopore and axial mesoderm from the early-gastrula (**O**) to early-neurula (**P**) stages. (**Q**, **R**) At the early-gastrula stage in shark, *wnt8* was expressed around the blastopore region (**Q**). At the early-neurula stage, expression was detected in the paraxial mesoderm and posteriorly (**R**). Scale bars, 500 µm.

**
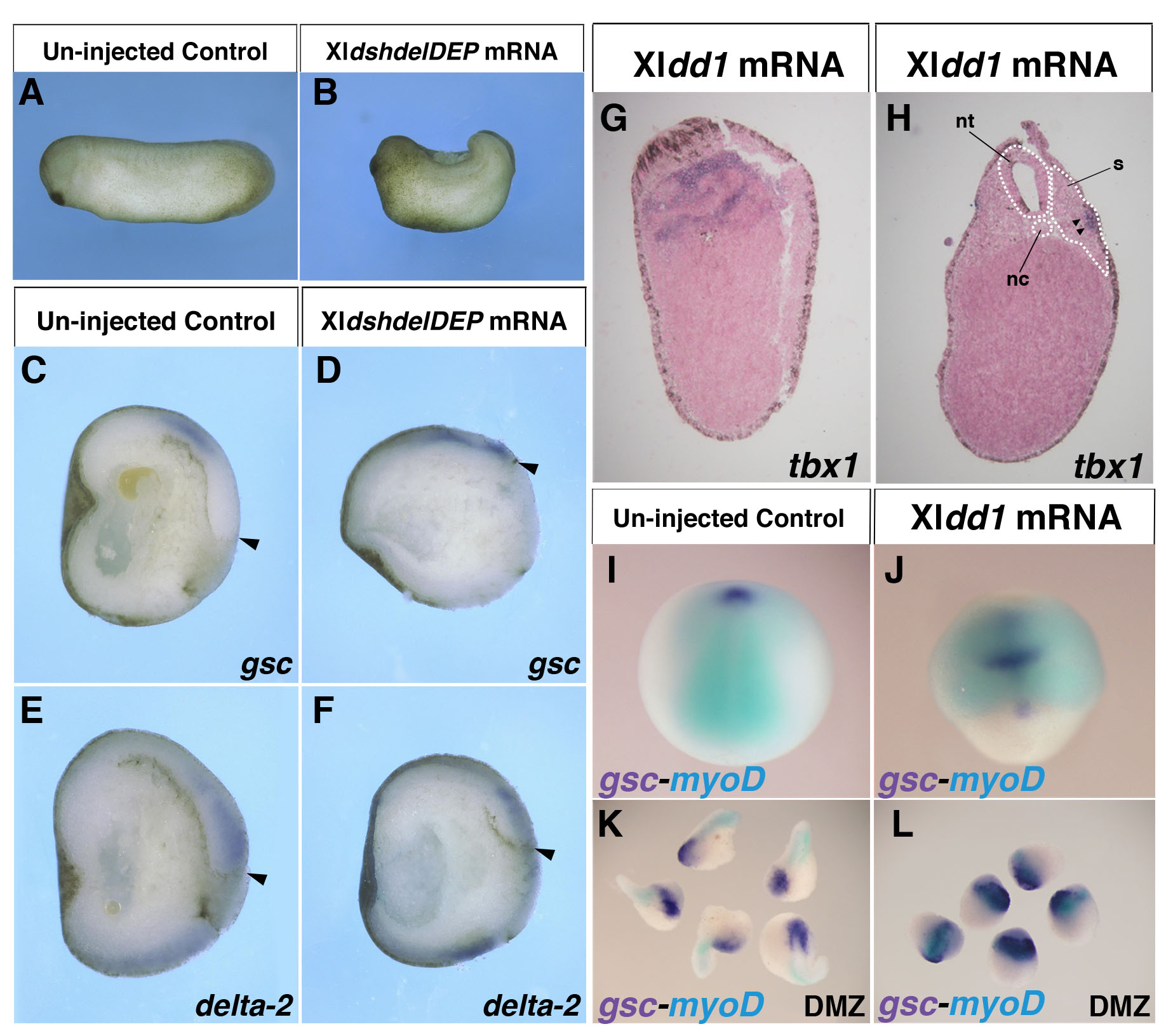
**

Figure S4. Suppression of the Wnt/PCP-signaling pathway in *Xenopus* embryos (see Figure 3) **(A**, **B)** Inhibition of the Wnt/PCP pathway. Control embryos showed well-defined head/trunk/tail mesodermal domains (**A**; n = 43, 98%), whereas those injected with 400 pg/cell Xl*dshdelDEP* mRNA at the four-cell blastomere stage showed loss of convergence and extension (**B**; n = 75, 61%). (**C**, **D**) Expression of *gsc* was normally detected in the migrating dorsal mesodermal cells, far from the blastopore lip (**C**; black arrowhead; n = 10, 100%). In 400 pg/cell Xl*dshdelDEP* mRNA–injected embryos, the expression domain of *gsc* remained around the blastopore (**D**; n = 17, 71%). In control embryos, *delta-2* was expressed in the posterior part of the dorsal mesoderm (**E**; n = 12, 100%). In 400 pg/cell Xl*dshdelDEP* mRNA–injected embryos, *delta-2* was expressed around the blastopore (**F**; n = 19, 74%). (**G**, **H**) In embryos injected with 400 pg/cell Xl*dd1* mRNA at the four-cell blastomere stage, *tbx1* expression was detected in the pharynx (**G**; n = 2, 100%) and somite (**H**; n = 2, 100%). nt, neural tube; s, somite; nc, notochord. The dotted lines indicate the neural tube, somites and notochord, respectively. (**I**, **J**) Double *in situ* hybridization of *gsc* (purple) and *myoD* (green) in stage 13 control (**I**; n = 11, 100%) and 400 pg/cell Xl*dd1* mRNA–injected embryos (**J**; n = 12, 83%). (**K, L**) DMZ assay and *in situ* hybridization for *gsc* (purple) and *myoD* (green). (**K**) In the control DMZ, the two markers did not overlap (n = 20, 100%). (**L**) In the 400 pg/cell Xl*dd1* mRNA–injected DMZ, *gsc* and *myoD* largely overlapped (n = 35, 94%).

**
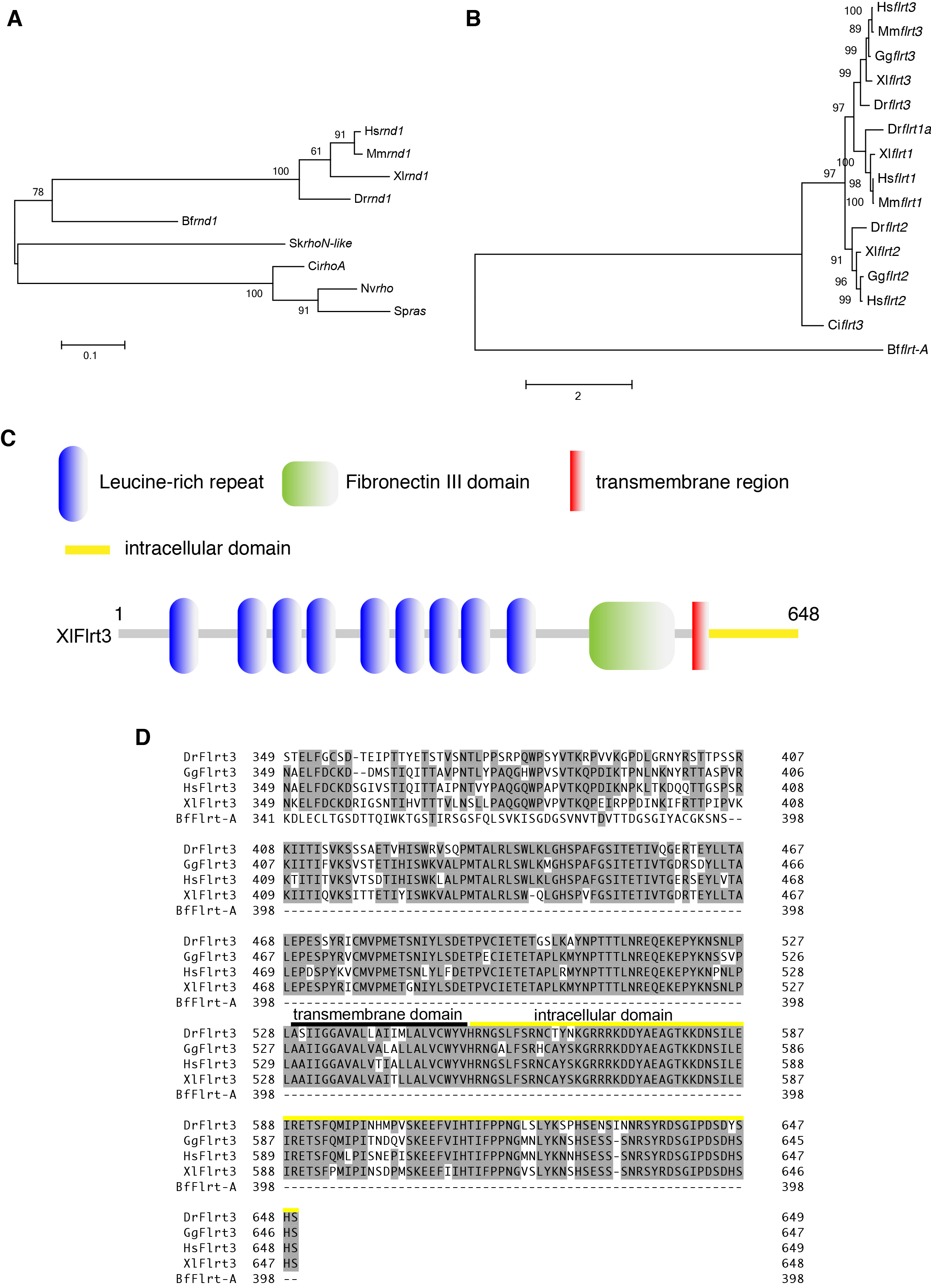
**

Figure S5. Flrt3 evolved in the vertebrate lineage (see Figure 4)

(**A**) Phylogenetic tree of *rnd1* genes: Hs*rnd1* (NP_055285), Mm*rnd1* (NP_766200), Xl*rnd1* (NP_001080385), Dr*rnd1* (NP_001037861), Bf*rnd1* (XP_002599495), Sk*rhoN-like* (XP_006821070), Ci*rhoA* (NP_001027690), Nv*rho* (XP_001631528), and Sp*ras* (NP_001229582). (**B**) Phylogenetic tree of *flrt3* genes: Hs*flrt3* (AAQ88685), Mm*flrt3* (NP_848469), Gg*flrt3* (XP_004940218), Xl*flrt3* (NP_001089030), Dr*flrt3* (NP_001243575), Dr*flrt1a* (XP_005168711), Xl*flrt1* (NP_001086411), Hs*flrt1* (NP_037412), Mm*flrt1* (NP_958813), Dr*flrt2* (NP_001093513), Xl*flrt2* (CAE54087), Gg*flrt2* (XP_003641353), Hs*flrt2* (AAI43937), Ci*flrt3* (XP_002120213.1), and Bf*flrt-A* (XP_002589584). (**C**) Protein domain structure of *Xenopus* Flrt3. (**D**) Alignment of Flrt3 proteins. The grey shading in the alignment indicates conserved amino acids. In vertebrates, Flrt3 possesses a transmembrane domain and an intracellular domain that binds to Rnd1 to promote cadherin degradation. However, the amphioxus protein (BfFlrt-A) does not contain these sequences, suggesting that the interaction between Flrt3 and Rnd1 originated in the vertebrate lineage. Hs, *Homo sapiens*; Mm, *Mus musculus*; Xl, *Xenopus laevis*; Dr, *Danio rerio*; Bf, *Branchiostoma floridae*; Sk, *Saccoglossus kowalevskii*; Ci, *Ciona intestinalis*; Nv, *Nematostella vectensis*; Sp, *strongylocentrotus purpuratus*; Gg, *Gallus gallus*. The sequences with accession numbers were obtained from NCBI (http://www.ncbi.nlm.nih.gov).


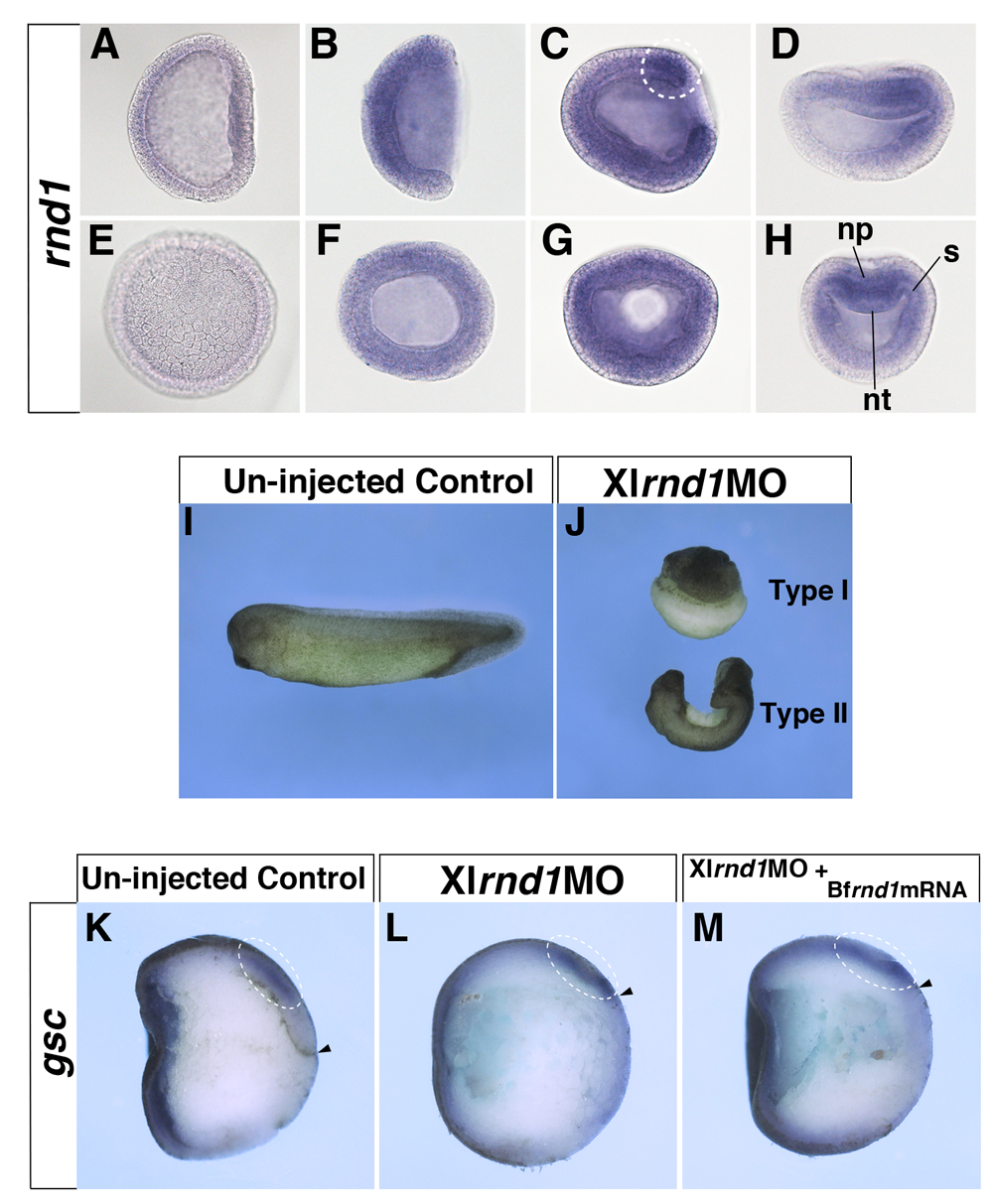


Figure S6. Flrt3-Rnd1 system is essential for mesoderm formation in

vertebrates (see Figure 3).

(**A–H**) *In situ* hybridization of *rnd1* in amphioxus. Side views at the early-gastrula (**A**), mid-gastrula (**B**), late-gastrula (**C**), and early-neurula (**D**) stages. White dotted line indicates the dorsal mesodermal expression of *rnd1*. Blastopore views at the early-gastrula (**E**), mid-gastrula (**F**), late-gastrula (**G**), and early-neurula (**H**) stages. np, neural plate; nt, notochord. Morphology of larval stage control embryos (**I**) and embryos injected with 0.2 mmol/cell Xl*rnd1*-MO (J). Type I embryos showed a severe gastrulation defect (n = 26, 15%), and type II embryos had a defect in convergence and extension (n = 26, 27%). (**K–M**) *gsc* expression in control (n = 10, 100%) and 0.2 mmol/cell Xl*rnd1*-MO-injected embryos (n = 18, 83%) and in embryos co-injected with 0.2 mmol/cell Xl*rnd1*-MO and 200 pg/cell Bf*rnd1* mRNA (n = 17, 100%). The dotted white line indicates the *gsc* expression pattern and the arrowhead indicates the blastopore lip.

| Gene | Accession number | Expression (early gastrula) | Expression (late-gastrula) | Ref |
| --- | --- | --- | --- | --- |
| *Mmgsc1* | NM_010351 | Primitive streak | Head mesoderm | [1] |
| *Gggsc1* | NM_205331 | Hensen’s node | Head mesoderm | [2] |
| *Xlgsc* | M81481 | Spemann’s organizer | Head mesoderm | [3] |
| *Drgsc1* | NM_131017 | Shield | Head mesoderm | [4] |
| *Bfgsc* | AF281674 | Nascent mesoderm | Notochord, presumptive chordal mesoderm | [5] |
| *Mmdelta1* | AAR30869 | No expression | Somites, PSM | [6] |
| *Mmdelta3* | NM_007866 | Pan mesoderm | Primitive streak, Somites, PSM | [7] |
| *Ggdelta1* | NP_990304 | Pan mesoderm | Primitive streak, Somite, PSM | [8] |
| *Xldelta2* | NM_001086082 | Pan mesoderm | Somite, PSM | [9] |
| *DrdeltaC* | ABL14114 | Pan mesoderm | Somites, PSM | http://zfin.org |
| *DrdeltaD* | AF426384_1 | Pan mesoderm | Somites, PSM | http://zfin.org |
| *Bfdelta* | XM_002590025 | 1st somites, posterior mesoderm | Somites, tail bud | [10] |
| *Mmwnt8a* | NP_033316 | Pan mesoderm | Pan mesoderm | [11]  http://www.informatics.jax.org |
| *Ggwnt8c* | NP_035850 | Pan mesoderm | Somites, PSM | http://geisha.arizona.edu/geisha/index.jsp |
| *Ggwnt8b* | XP_426508 | Pan mesoderm | No data | http://geisha.arizona.edu/geisha/index.jsp |
| *Xlwnt8a* | NP_001081637 | Pan mesoderm | Somites, PSM | http://www.xenbase.org/entry/ |
| *Drwnt8a* | NP_571021 | Pan Mesoderm | Somites, PSM | [12] |
| *Drwnt8b* | NP_571034 | No data | Somites | [12] |
| *Bfwnt8* | AF190470_1 | Pan mesoderm | Somites, tail bud | [13] |
| *Mmdkk1* | AAC02426 | No expression | Head mesoderm | http://www.informatics.jax.org |
| *Ggdkk1* | XP_421563 | Hensen’s node, Primitive streak | Pan mesoderm | http://geisha.arizona.edu/geisha/index.jsp |
| *Xldkk1* | ABM83148 | Spemann’s organizer | Pan mesoderm | http://www.xenbase.org/entry/ |
| *Drdkk1* | BAA82135 | Pan mesoderm | Pan mesoderm | http://zfin.org |
| *Bfdkk1/2/4* | ABG34307 | Pan mesoderm | Pan mesoderm | [13] |

Table S1. Summary of expression pattern of genes in Figure S2 (see Figure 2).

The genes expressed in the mesoderm were selected and only mesodermal expression domains during gastrulation were described.

**References**

1. Blum M, Gaunt SJ, Cho KW, Steinbeisser H, Blumberg B, Bittner D et al. Gastrulation in the mouse: the role of the homeobox gene goosecoid. Cell. 1992;69(7):1097-106.

2. Izpisua-Belmonte JC, De Robertis EM, Storey KG, Stern CD. The homeobox gene goosecoid and the origin of organizer cells in the early chick blastoderm. Cell. 1993;74(4):645-59.

3. Cho KW, Blumberg B, Steinbeisser H, De Robertis EM. Molecular nature of Spemann's organizer: the role of the Xenopus homeobox gene goosecoid. Cell. 1991;67(6):1111-20.

4. Griffin K, Patient R, Holder N. Analysis of FGF function in normal and no tail zebrafish embryos reveals separate mechanisms for formation of the trunk and the tail. Development. 1995;121(9):2983-94.

5. Neidert AH, Panopoulou G, Langeland JA. Amphioxus goosecoid and the evolution of the head organizer and prechordal plate. Evol Dev. 2000;2(6):303-10.

6. Bettenhausen B, Hrabe de Angelis M, Simon D, Guenet JL, Gossler A. Transient and restricted expression during mouse embryogenesis of Dll1, a murine gene closely related to Drosophila Delta. Development. 1995;121(8):2407-18.

7. Dunwoodie SL, Henrique D, Harrison SM, Beddington RS. Mouse Dll3: a novel divergent Delta gene which may complement the function of other Delta homologues during early pattern formation in the mouse embryo. Development. 1997;124(16):3065-76.

8. Bothe I, Dietrich S. The molecular setup of the avian head mesoderm and its implication for craniofacial myogenesis. Dev Dyn. 2006;235(10):2845-60.

9. Peres JN, McNulty CL, Durston AJ. Interaction between X-Delta-2 and Hox genes regulates segmentation and patterning of the anteroposterior axis. Mech Dev. 2006;123(4):321-33.

10. Rasmussen SL, Holland LZ, Schubert M, Beaster-Jones L, Holland ND. Amphioxus AmphiDelta: evolution of Delta protein structure, segmentation, and neurogenesis. Genesis. 2007;45(3):113-22.

11. Bouillet P, Oulad-Abdelghani M, Ward SJ, Bronner S, Chambon P, Dolle P. A new mouse member of the Wnt gene family, mWnt-8, is expressed during early embryogenesis and is ectopically induced by retinoic acid. Mech Dev. 1996;58(1-2):141-52.

12. Kelly GM, Greenstein P, Erezyilmaz DF, Moon RT. Zebrafish wnt8 and wnt8b share a common activity but are involved in distinct developmental pathways. Development. 1995;121(6):1787-99.

13. Yu JK, Satou Y, Holland ND, Shin IT, Kohara Y, Satoh N et al. Axial patterning in cephalochordates and the evolution of the organizer. Nature. 2007;445(7128):613-7.
